# Reversible insulin resistance helps Bactrian camels survive fasting

**DOI:** 10.1038/s41598-021-98234-y

**Published:** 2021-09-22

**Authors:** Fucheng Guo, Rendalai Si, Quanyun Li, Le Hai, Li Yi, Jing He, Liang Ming, Rimutu Ji

**Affiliations:** 1 Key Laboratory of Dairy Biotechnology and Bioengineering, Ministry of Education, College of Food Science and Engineering, Inner Mongolia Agricultural University, Hohhot, 010018 China; 2 Camel Research Institute of Inner Mongolia, Alxa, 737300 China

**Keywords:** Developmental biology, Zoology

## Abstract

Camels have hunger tolerance and can adapt to the severe environment of the desert. Through the comparison of insulin signalling pathway genes in different tissues in different eating periods (feeding, fasting and recovery feeding), it was found that IRS1, PIK3CB, PIK3R1 and SLC2A4 expression was significantly downregulated in the fore hump and hind hump during the fasting period. In addition, there was no difference in serum insulin levels among the three stages. However, the serum leptin and adiponectin levels decreased significantly during fasting. Additionally, insulin tolerance tests during the three stages showed that camels were insensitive to insulin during fasting. Further study of the serum metabolites showed that serum branched-chain and aromatic amino acid levels increased during the fasting period. Finally, analysis of microbial diversity in camel faeces at different stages showed that during the fasting period, the proportion of *Firmicutes* and *Actinobacteria* increased, while that of *Bacteroides* and the butyrate-producing bacterium *Roseburia* decreased. The results of this study show that fasting is accompanied by changes in the activation of insulin pathways in various camel tissues, normal insulin levels, and increased lipolysis and insulin resistance, which return to normal after eating.

## Introduction

Desert camels (Camelus bactrianus) can adapt to severe environments lacking food and water resources in arid and semiarid areas, regulate their body fluid balance and body temperature under stress, and survive longer than other ruminants under conditions of scarce and poor-quality food^1^. Camels can also reduce their feed intake when necessary^2^. Bactrian camels are one of the few large domestic animals that can adapt to the environment of the Gobi Desert^3^ because they can store a large amount of energy in the form of fat in the hump and abdomen so that they can prolong survival when food is scarce^4^. Camels can also reduce their metabolic rate when facing famine^2^. Furthermore, according to our previous research^3^, this approach seems to be the most efficient choice. These characteristics of camels make them more able to resist the impact of hunger than other livestock and to cope with the severe natural environment in deserts. Therefore, the domestic Bactrian camel was the major transport carrier on the ancient Silk Road. Camels usually do not receive food for long periods of time during transportation that lasts for months or more but can still tenaciously reach their destination. It has been reported that camels can maintain normal body function after fasting for 4^5^, 7^6^ and 11^4^ days. In cold winters, wild two-humped camels (Camelus ferus) in the desert may not be able to obtain food for dozens of days. In addition, domestic Bactrian male camels rarely eat and drink for one month when they courtship, which may be related to hormone regulation, but it still shows their unique anti-hunger ability.

According to the current research, there are two main reasons for camels’ amazing hunger tolerance. One is that they can store a large amount of fat in their bodies^3,4^, and the other is that they can reduce their metabolism when hungry^3^. When food is abundant, camels accumulate a large amount of fat in their hump, and when famine comes, camels overcome difficulties through fat decomposition. However, there are few reports on the specific molecular mechanisms of camel resistance to famine. Therefore, the molecular mechanism of camel starvation tolerance is worthy of attention and in-depth study. In this study, we compared metabolic and transcriptional changes in the three stages of feeding, fasting, and recovery feeding.

## Results

### Changes in body weight

**Figure 1:**
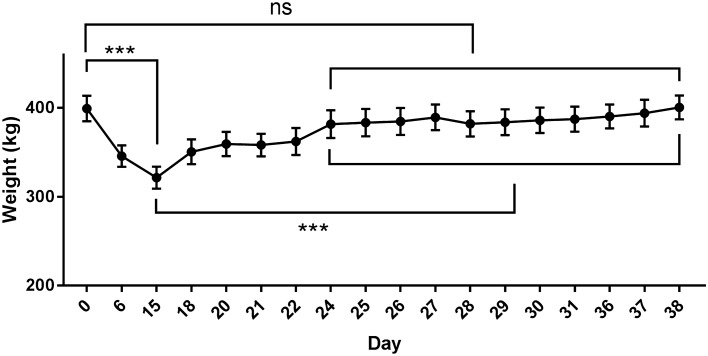
Changes in body weight of camels during the experiment (statistical analyses were performed using multiple unpaired t tests, ***p < 0.001; all error bars represent ± SEM).

On the 0th day of the experiment, the body weight of the camel was 399.33 ± 35.41 kg, and on the 15th day of fasting, the camel's weight was 321.42 ± 30.38 kg. At this time, the camel's body weight decreased by 19.51%, which was significantly lower than that on the 0th day (*p* < 0.001).There was no significant difference in camel body weight between the 24th day and the 0th day. For the camel to fully recover, the recovery period lasted until the 38th day, when the body weight was basically the same as that on the 0th day (Fig. 1).

### Insulin pathway gene expression

**Figure 2:**
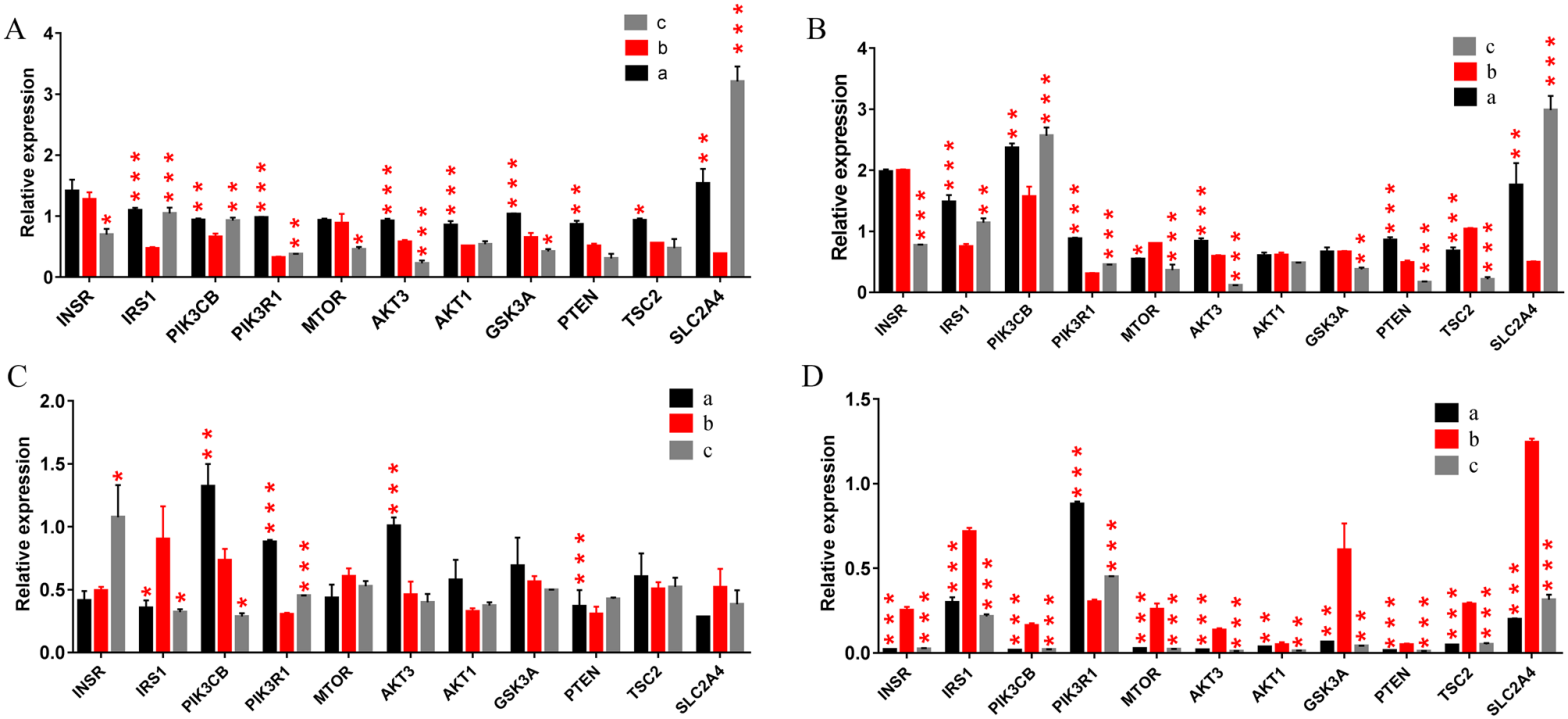
qRT-PCR results for genes in tissues of Bactrian camels reported as relative expression. a: feeding period, b: fasting period, c: recovery feeding period. (**A**) Fore hump, (**B**) hind hump, (**C**) subcutaneous fat, (**D**) biceps femoris muscle. Statistical determinations were made using one-way ANOVA (*p < 0.05, **p < 0.01, ***p < 0.001). Red asterisks indicate significant differences compared to b.

The results (Fig. 2) showed that in fore hump and hind hump, insulin receptor substrate-1 (IRS1), PIK3CB, PIK3R1 and solute carrier family 2 member 4 (SLC2A4) expression was upregulated in feeding period and recovery feeding period but downregulated in fasting period. At the same time, in subcutaneous fat, at fasting period, IRS1 expression was upregulated, but PIK3R1 expression was downregulated. It is worth noting that in biceps femoris muscle, in fasting period, INSR, IRS1, PIK3CB, MTOR, AKT3, GSK3A, PTEN, TSC2 and SLC2A4 expression was upregulated, and only PIK3R1 expression was downregulated.

### Serum phenotypes and insulin resistance test

**Figure 3:**
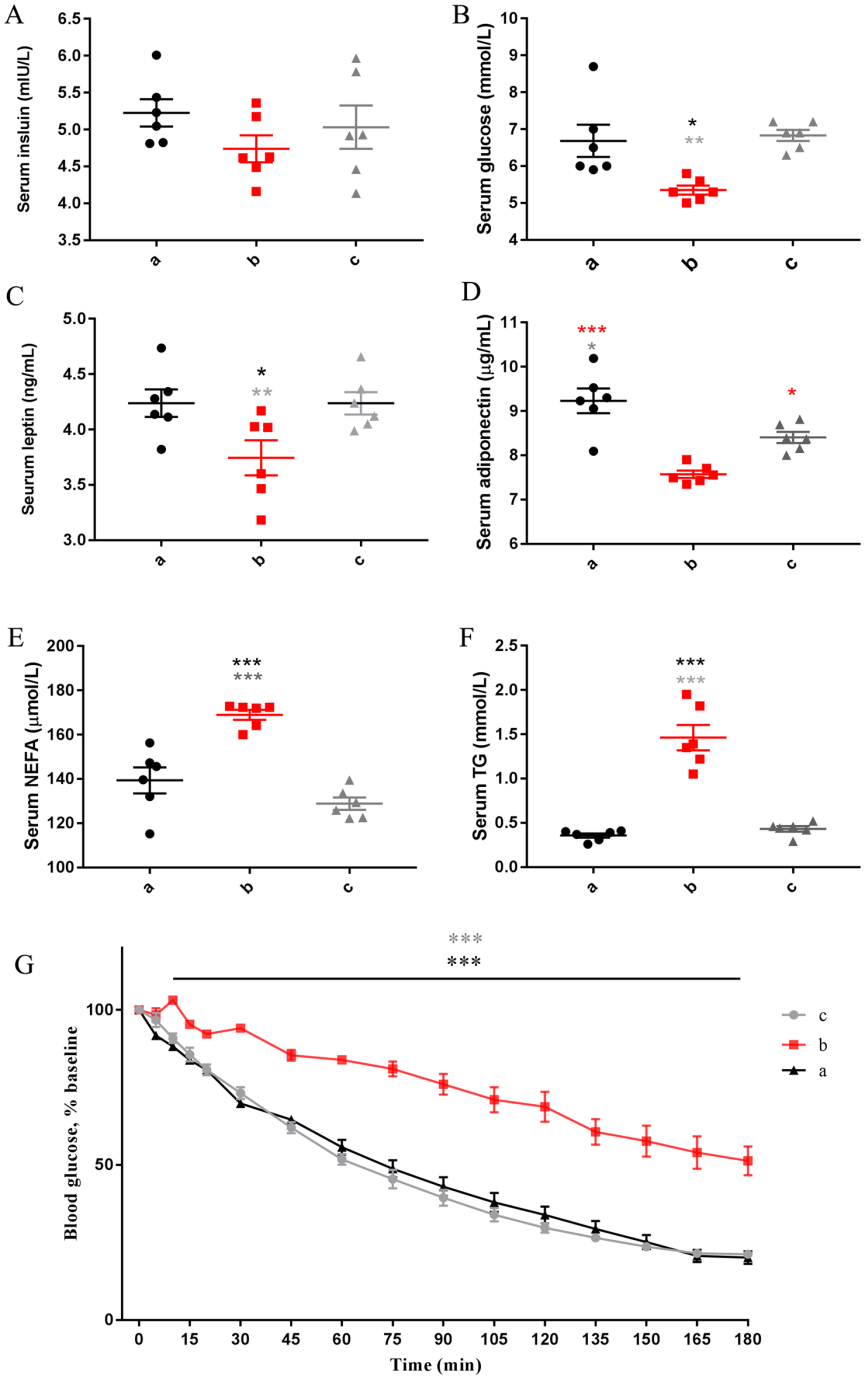
(**A**) Serum insulin levels. (**B**) Serum glucose levels. (**C**) Serum leptin levels. (**D**) Serum adiponectin levels. (**E**) Serum nonesterified fatty acid (NEFA) levels. (**F**) Serum triglyceride (TG) levels. Statistical analysis was performed by one-way ANOVA with Tukey’s multiple comparison post-test (*p < 0.05, **p < 0.01, ***p < 0.001). (**G**) Insulin tolerance tests (ITTs). Statistical analyses were performed using multiple unpaired t tests (*p < 0.05, **p < 0.01, ***p < 0.001). a: Feeding period, b: fasting period, c: recovery feeding period. Black, red and grey asterisks indicate significant differences compared to a, b and c, respectively. All error bars represent ± SEM.

There was no difference in serum insulin levels among the three periods (Fig. 3A). However, the blood glucose (Fig. 3B) and serum leptin (Fig. 3C) and adiponectin (Fig. 3D) levels in fasting period were significantly lower than those in feeding and recovery feeding period. Additionally, the serum nonesterified fatty acid (NEFA, Fig. 3E) level in fasting period was higher than that in feeding and recovery feeding period. Notably, triglyceride (TG, Fig. 3F) levels increased significantly in fasting period. Finally, the insulin tolerance test (ITT) results in the three stages showed that camels were insensitive to insulin in stage b (Fig. 3G).

### Metabolomics analysis of serum

**Figure 4:**
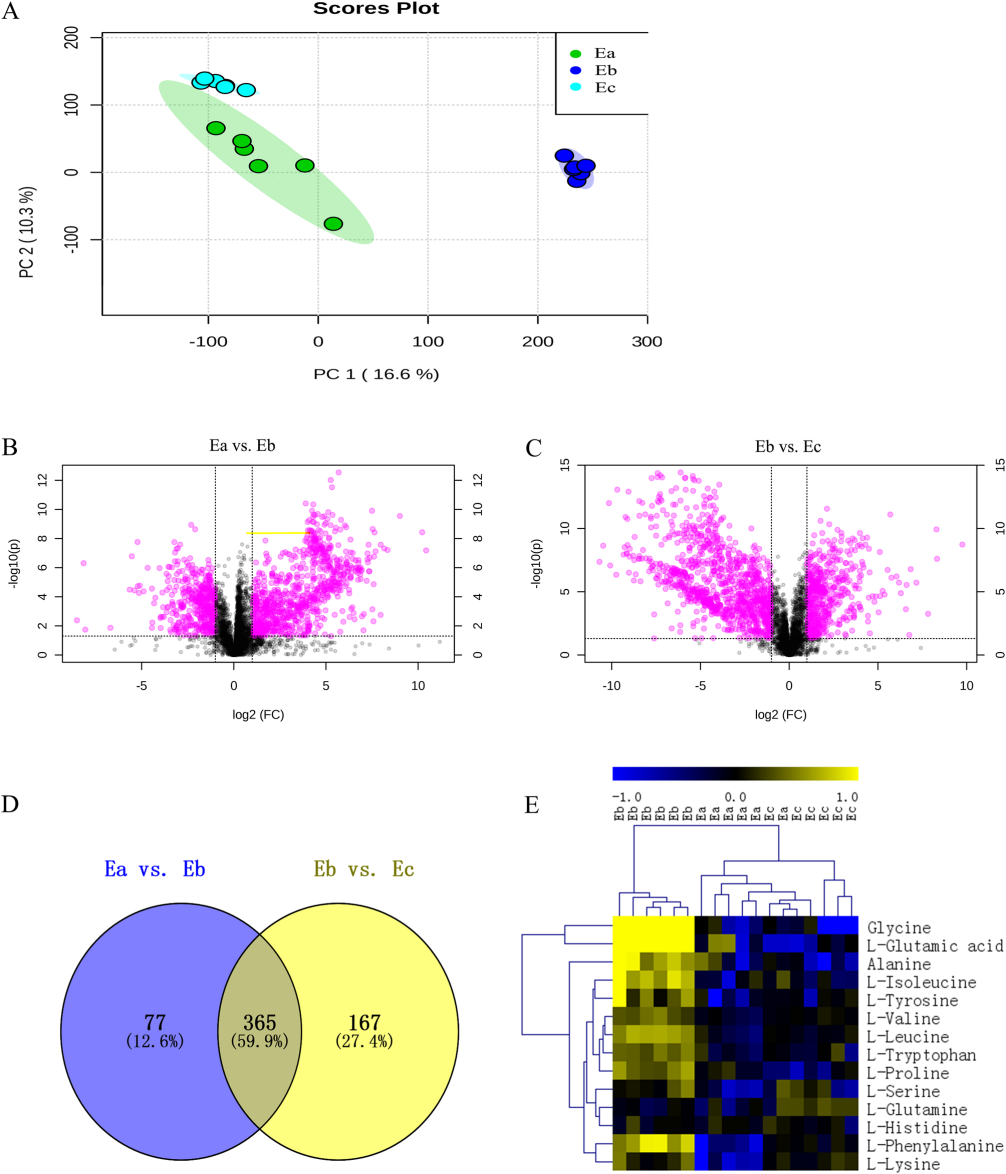
(**A**) Principal component analysis (PCA) of serum metabolite levels. (**B**) Volcano map of DEMs in E in stage a vs. stage b (False Discovery Rate (FDR) < 0.05, |fold change|> 2). (**C**) Volcano map of DEMs in E in stage b vs. stage c (False Discovery Rate (FDR) < 0.05, |fold change|> 2 ). (**D**) Venn analysis map of serum DEMs. (**E**) Cluster heat map of serum branched-chain amino acid and aromatic amino acid levels. (**E**) Serum, DEMs: differentially expressed metabolites, a: feeding period, b: fasting period, c: recovery feeding period.

First, the serum metabolites in three stages were analysed by principal component analysis (PCA). The results showed that serum in fasting period was significantly separated from serum in feeding and recovery feeding period (Fig. 4A). Furthermore, to investigate the changes in serum metabolites before and after fasting, we compared and analysed the differences. A total of 445 differentially expressed metabolites (DEMs) were identified in serum in feeding vs. fasting period, of which 298 and 147 metabolites in serum in feeding and fasting period were enriched, respectively (Table 1, Fig. 4B). In addition, a total of 532 DEMs were identified from serum in fasting vs. recovery feeding period, of which 183 and 349 metabolites in serum in fasting and recovery feeding period were enriched, respectively (Table 1, Fig. 4C).

**Table 1:** Comparison of different metabolites in serum (false discovery rate (FDR) < 0.05, variable importance in the projection (VIP) > 1, fold change (FC) > 2, or FC < 0.5, differentially expressed metabolites (DEMs), serum (E), a: feeding period, b: fasting period, c: recovery feeding period).

Items	Ea versus Eb	Eb versus Ec
No. of DEMs	445	532
No. of upregulated DEMs	298 (Ea) 147 (Eb)	183 (Eb) 349 (Ec)

The above two groups of DEMs were analysed by Venn analysis. A total of 365 overlapping metabolites were obtained, which were involved in the whole process of stage a to stage b and stage b to stage c changes in serum (Fig. 4D). Furthermore, the KEGG pathways of these 365 DEMs were analysed, and 32 KEGG pathways were identified (*P* adjusted < 0.05, Supplementary Table 1, 2). These pathways are mainly involved in amino acid metabolism, biosynthesis of other secondary metabolites, carbohydrate metabolism, membrane transport, metabolism of cofactors and vitamins, endocrine and metabolic diseases, lipid metabolism and other biological processes. The top 5 KEGG pathways were secondary bile acid biosynthesis, central carbon metabolism in cancer, protein digestion and absorption, phenylalanine metabolism and aminoacyl-tRNA biosynthesis. In addition, the analysis of branched-chain amino acids (BCAAs) and aromatic amino acids (AAAs) in serum showed that glycine, glutamic acid, alanine, isoleucine, tyrosine, valine, leucine, tryptophan, proline and phenylalanine levels were significantly upregulated in serum in fasting period (Fig. 4E).

### Analysis of microbial diversity of faeces

**Figure 5:**
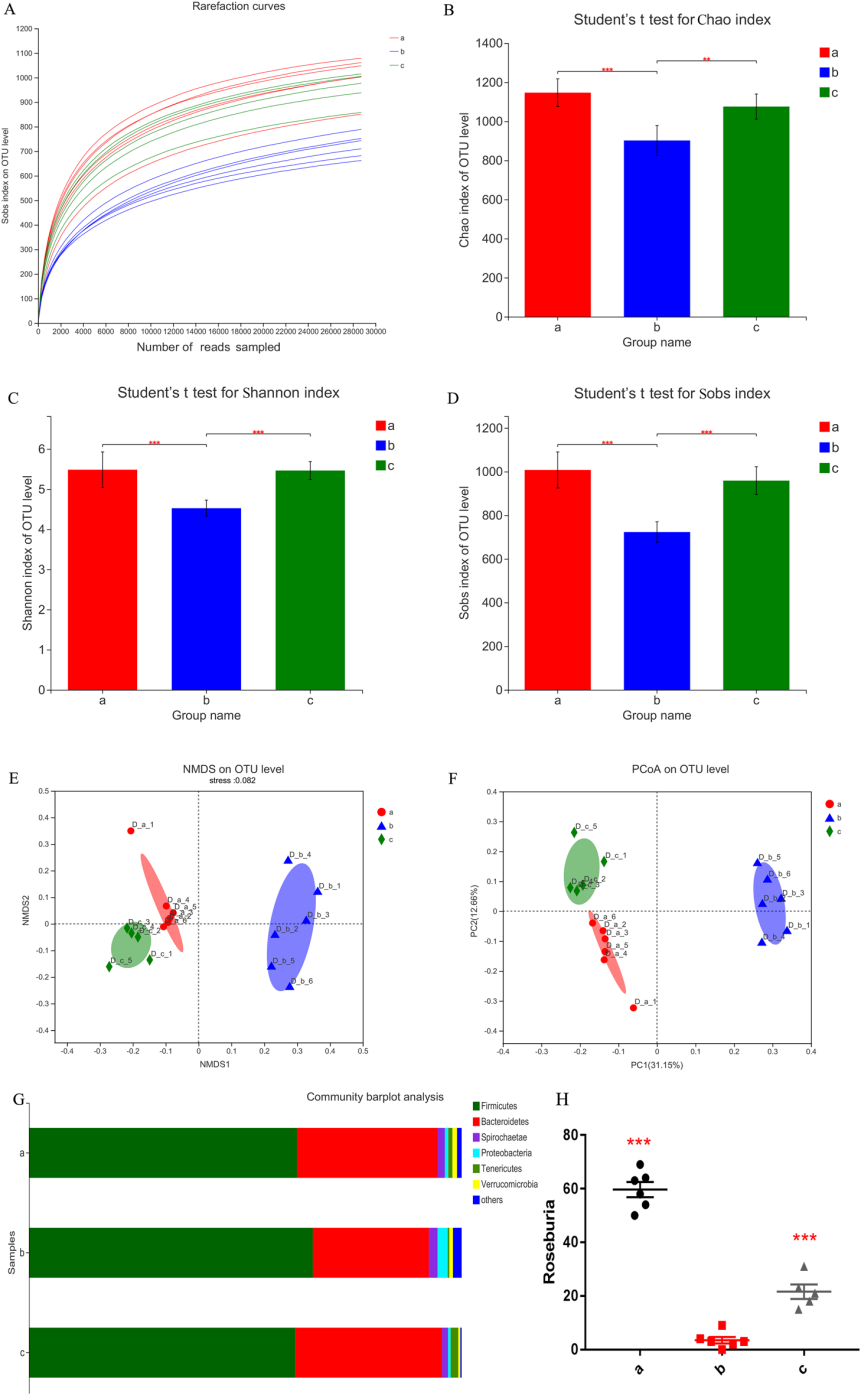
(**A**) Faecal sample rarefaction curves. (**B**) The Chao estimator of camel faecal flora in different dietary stages. (**C**) The Shannon diversity index of camel faecal flora in different dietary stages. (**D**) The observed richness (Sobs) of camel faecal flora in different dietary stages. Statistical analysis was performed by one-way ANOVA with Tukey’s multiple comparison post-test, *0.01 < *P* ≤ 0.05, ** 0.001 < *P* ≤ 0.01, *** *P* ≤ 0.001. (**E**) Nonmetric multidimensional scaling (NMDS) ordination plots of faecal bacterial communities. (**F**) Principal coordinate analysis of microbial community membership. (**G**) Faecal microbial composition at the phylum level. (**H**) Richness of Roseburia in the faeces of Bactrian camels. Statistical analysis was performed by one-way ANOVA with Tukey’s multiple comparison post-test (*p < 0.05, **p < 0.01, ***p < 0.001). a: Feeding period, b: fasting period, c: recovery feeding period.

Across all 24 faecal samples, 1,453,555 high-quality sequences were classified as bacteria, with an average length of 433 bp (Supplementary Table 3). The rarefaction curve (average curve of each group) results showed that most of the microbial diversity was fully captured (Fig. 5A). The statistical estimates of α-diversity showed that the Chao1 diversity index, Shannon estimator and Sobs diversity index of faeces in fasting period were significantly lower than those in feeding and recovery feeding period (Fig. 5B–D).

**Figure 6:**
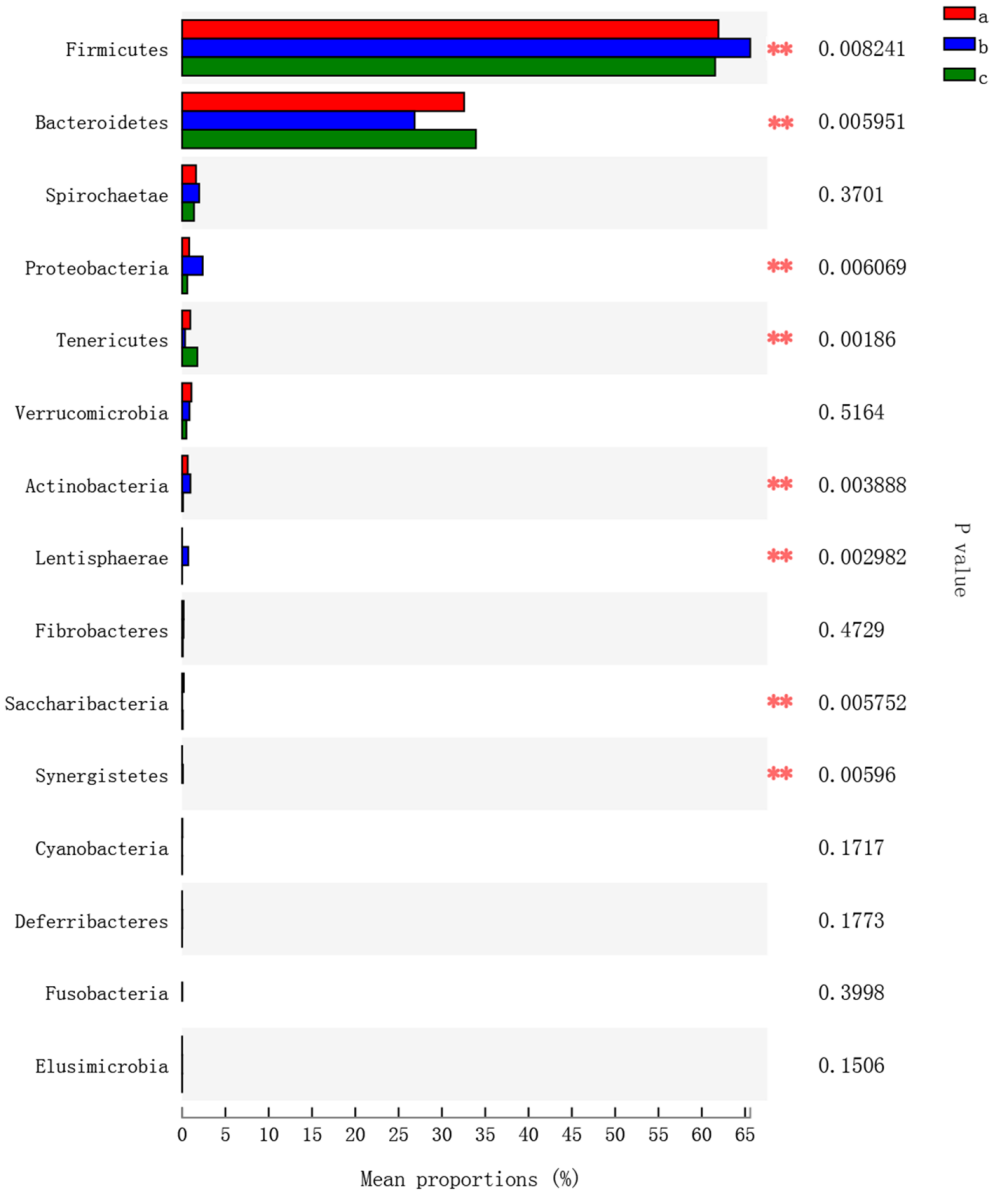
Statistical analysis of dominant phyla in faeces. a: Feeding period, b: fasting period, c: recovery feeding period. Statistical analysis was performed by one-way ANOVA with Tukey’s multiple comparison post-test, *0.01 < *P* ≤ 0.05, ** 0.001 < *P* ≤ 0.01.

The results of the nonmetric multidimensional scaling (NMDS, Fig. 5E) ordination plot and principal coordinates analysis (PCoA, Fig. 5F) showed that the bacterial communities in faeces were obviously distinct in the feeding, fasting and recovery feeding period. To further examine the bacterial groups in different dietary stages, the bacterial groups with a relative abundance of more than 1% were classified and analysed. At the phylum level, *Firmicutes* and *Bacteroidetes* were the most predominant phyla in the faeces (Fig. 5G). Furthermore, the Kruskal–Wallis H test was used to analyse the community abundance data of each faecal sample. The results showed that in the faeces in fasting period, the proportions of *Firmicutes*, *Proteobacteria*, *Actinobacteria* and *Lentisphaerae* increased, while the proportions of *Bacteroidetes* and *Tenericutes* decreased (Fig. 6, *P* < 0.01).

**Figure 7:**
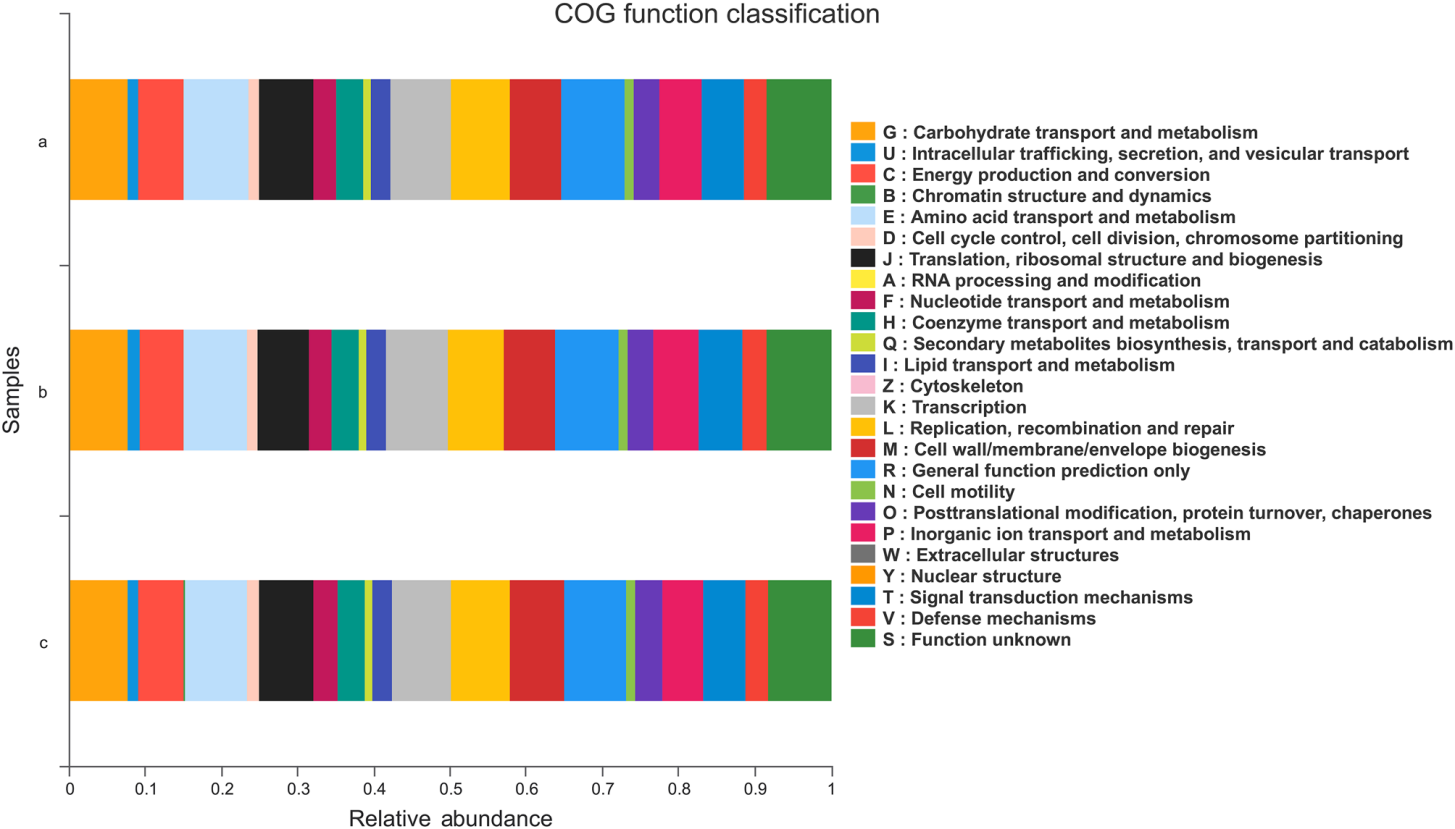
Statistical analysis of dominant phyla in faeces. a: Feeding period, b: fasting period, c: recovery feeding period. Statistical analysis was performed by one-way ANOVA with Tukey’s multiple comparison post-test, *0.01 < *P* ≤ 0.05, ** 0.001 < *P* ≤ 0.01.

To explore the function of microorganisms in the samples, we used PICRUSt for functional analysis. Comparing the predicted clusters of orthologous groups of proteins (COG) and KEGG functions of the faeces in fasting period with those of the faeces in feeding and recovery feeding period, we found that the faeces in fasting period had strong functions of carbohydrate metabolism, amino acid metabolism, lipid transport and metabolism, biosynthesis and catabolism of secondary metabolites, inorganic ion transport and metabolism (Fig. 7, *P* < 0.01). However, the G protein-coupled receptors, apoptosis, ether lipid metabolism, nucleotide metabolism, and cell division energy metabolism pathways the faeces in fasting period were significantly downregulated (Supplementary Table 4, *P* < 0.01).

## Discussion

Although all animals experience feeding and fasting periods, the species that are most interesting are those that can survive fasting for a long period of time. How to fast for a long time by relying only on endogenous resources has been a hot topic for researchers for a long time^7^. At present, there are few studies on camel fasting and hunger tolerance, and the mechanism remains to be further clarified. In this study, qRT-PCR, 16S rDNA sequencing and metabonomics techniques, combined with phenotypic tests, were used to reveal the strategies that Bactrian camels use to cope with fasting.

Stage-dependent changes in the expression of key insulin signalling genes were observed in fore hump (Fig. 2A), hind hump (Fig. 2B), subcutaneous fat (Fig. 2C), and biceps femoris muscle (Fig. 2D). In fore hump and hind hump, IRS1, PIK3CB, PIK3R1 and SLC2A4 expression was significantly downregulated in stage b. In contrast, in biceps femoris muscle, INSR, IRS1, PIK3CB, MYOR, AKT1, AKT3, GSK3A, PTEN, TSC2 and SLC2A4 expression was significantly upregulated in fasting period. This result suggests that hump and muscle tissue may mediate different insulin states in fasting period. Interestingly, in subcutaneous fat, IRS1 expression was upregulated in fasting period, while PIK3R1 expression was downregulated.

IRS1 plays an important role in controlling the dynamic balance of growth and nutrition and acts as an on/off switch to transduce insulin action^8,9^. In addition, IRS1 is closely related to PI3K (PIK3CB and PIK3R1) and AKT (AKT1, AKT2 and AKT3) activation. Loss of IRS1 can cause PI3K inactivation and eventually lead to insulin resistance^10^. Insulin resistance is also associated with decreased expression of the insulin-sensitive glucose transporter 4 protein (GLUT4) encoded by SLC2A4^11^. GLUT4 is the main glucose transporter subtype in insulin-responsive tissues. Genetically engineered mice overexpressing the exogenous GLUT4 gene in skeletal muscle or adipose tissue exhibit increased insulin responsiveness and peripheral glucose utilization^12^. In genetic and experimental models of diabetes, high transporter levels enhance the ability to respond to insulin, thereby reversing the diabetes phenotype^13^. Therefore, we speculate that in fasting period, the insulin signalling pathway is inhibited in fore hump and hind hump, while it is enhanced in biceps femoris muscle, and its function in subcutaneous fat remains to be clarified. According to reports, the adipose tissue and muscles of brown bears exhibit insulin resistance during hibernation, which helps them survive the long winter^14^. Interestingly, the camel fore hump and hind hump exhibit insulin resistance during the fasting period, but the insulin sensitivity of the muscle seems to be enhanced. We speculate that the differences in strategies for coping with famine may be related to their respective living environments. Camels living in the Gobi Desert face food shortages not only in winter but also in other seasons. Therefore, camels do not rely on sleep to reduce the energy consumption of the whole body, as the brown bear does, but instead use the internal coordination of the body; camels therefore first guarantee the energy supply of more critical parts, such as muscles.

We initially assumed that prolonged fasting would induce hypoinsulinaemia and lead to loss of activation of the insulin pathway^15^. This may be the reason for the downregulation of the expression of the main insulin pathway genes of fore hump and hind hump in fasting period. Contrary to our expectations, insulin levels of all camels did not change at all three stages (Fig. 3A). This result is consistent with hibernating animals during the winter fasting period^16^. This further indicates that during fasting, the insulin sensitivity of hump tissue decreases, while that of muscle tissue increases. The level of blood glucose in stage b decreased significantly, which was closely related to fasting (Fig. 3B). Fasting reduces circulating leptin levels, while eating or obesity increases leptin levels^16–18^, which is consistent with our results (Fig. 3C). Adiponectin can enhance peripheral insulin sensitivity, which is negatively correlated with insulin resistance^19–24^. In addition, elevated serum NEFA levels can induce insulin resistance^25,26^. Therefore, serum adiponectin (Fig. 3D) and NEFA levels (Fig. 3E) support the decreased insulin sensitivity of camels in stage b.

In addition to controlling blood glucose, insulin also effectively inhibits lipolysis of adipose tissue^27^, which is a multistep process that promotes the triglycerides into glycerol and free fatty acids. However, the results showed that the serum triglyceride content of camels in fasting period was significantly increased (Fig. 3F), indicating active lipolysis. It has been reported that a high lipolysis rate is associated with reduced human insulin sensitivity^28^. Therefore, camels may have insulin resistance in stage b. For insulinaemia, the different activation of the insulin pathway at different stages indicates that the camel's response to insulin is different. To solve this problem directly, we conducted an ITT. The results showed insulin resistance in stage b camels (Fig. 3G). This finding also indicates that the downregulation of the expression of insulin pathway genes in F and H is positively correlated with insulin resistance. Collectively, these data suggest that Bactrian camels modulate states of reversible insulin responsiveness.

In view of the change in insulin sensitivity of camels caused by fasting, we then investigated its effects on metabolites in camel serum. PCA shows (Fig. 4A) that the differences between data sets are largely due to fasting, which is consistent with the trend of insulin pathway genes. In recent years, scientists have found that BCAAs^29–35^ and AAAs^36–38^ in blood are positively correlated with insulin resistance. This finding is consistent with our results (Fig. 4E). In stage b, serum BCAA (leucine, isoleucine and valine) and AAA (tyrosine, phenylalanine and tryptophan) levels increased significantly, which further indicated that camels may be in a state of insulin resistance during fasting.

One of the characteristics of the serum metabolites of individuals with insulin resistance is an increased serum BCAA level, which is related to the intestinal microflora and has rich potential for BCAA biosynthesis^39^. The increase of branched chain amino acids in insulin resistance may be related to the changes of peripheral amino acid metabolism, but intestinal microflora has been proved to be very important for the supply of leucine, isoleucine and valine (BCAA) in mammalian hosts. In addition, increasing evidence shows that there is a link between the intestinal microflora and metabolic health^40–43^. The insulin resistance phenotype is transferable through faecal microbiota transplantation (FMT)^44,45^. Therefore, we analysed the faecal microbial diversity of camels in different stages.

It has been reported that hydrolysis and fermentation of dietary polysaccharides by the intestinal microflora will produce a large number of monosaccharides and short-chain fatty acids (SCFAs)^46^, which can be absorbed and utilized as energy by the host, and obesity-associated microflora generate energy from the diet more effectively^47,48^. Through the study of humans and animals, it has been found that a higher ratio of *Firmicutes* to *Bacteroidetes* in the intestinal tract can result in more effective energy acquisition from the diet and may lead to obesity^49,50^, and at the same time, it will promote the increase of BCAA^51^. It was also reported that insulin-resistant subjects showed an increase in the relative abundances of *Firmicutes* and *Actinobacteria* and a decrease in the relative abundance of *Bacteroides*^52^. This finding is consistent with our results (Figs. 5G, 6), indicating that camels may exhibit insulin resistance during the fasting period, and the camel intestinal microflora is more efficient at obtaining energy from gastrointestinal contents to better survive through the fasting period.

Studies have shown that FMT from lean male donors significantly improved insulin sensitivity in male patients with metabolic syndrome and increased intestinal microbial diversity, including a significant increase in the abundance of butyrate-producing strains^46^. Compared with that in the intestinal flora of healthy people, the number of butyrate-producing bacteria in the intestinal flora of type 2 diabetes (T2DM) patients decreased; that is, the abundance of *Roseburia* decreased^41,42^. Our research shows that the *Roseburia* abundance of camels decreased significantly during the fasting period (Fig. 5H). Studies have shown that the increase of *Proteobacteria* will promote the synthesis of BCAA, which is consistent with our research (Fig. 6)^53^.Taken together, these results indicate that the intestinal flora of camels during the fasting period is similar to that of obese or diabetic patients, which may lead to insulin resistance.

It should be pointed out that the investigation of the genes involved in insulin signaling has importance, but does not define the real insulin signaling pathway, which depends on tyrosine kinase activity of insulin receptor, downstream tyrosine phosphorylation and serine phosphorylations. This is the limitation of this study, and will be explored in follow-up studies.

The results of this study showed that during the fasting period, the insulin sensitivity of fore hump and hind hump decreased, while that of muscle increased, but overall, the camels were in a state of insulin resistance, and lipolysis increased. Additionally, serum BCAA and AAA levels, as well as the abundances of *Firmicutes*, *Actinobacteria*, *Bacteroides* and *Roseburia*, can be used as candidate markers in camels for the diagnosis of insulin resistance; thus, these results provide directions for future research. Our findings reveal the molecular mechanism of camel tolerance to hunger and may provide a new animal model for human research on metabolic diseases.

## Materials and methods

### Animals and sample collection

**Figure 8:**
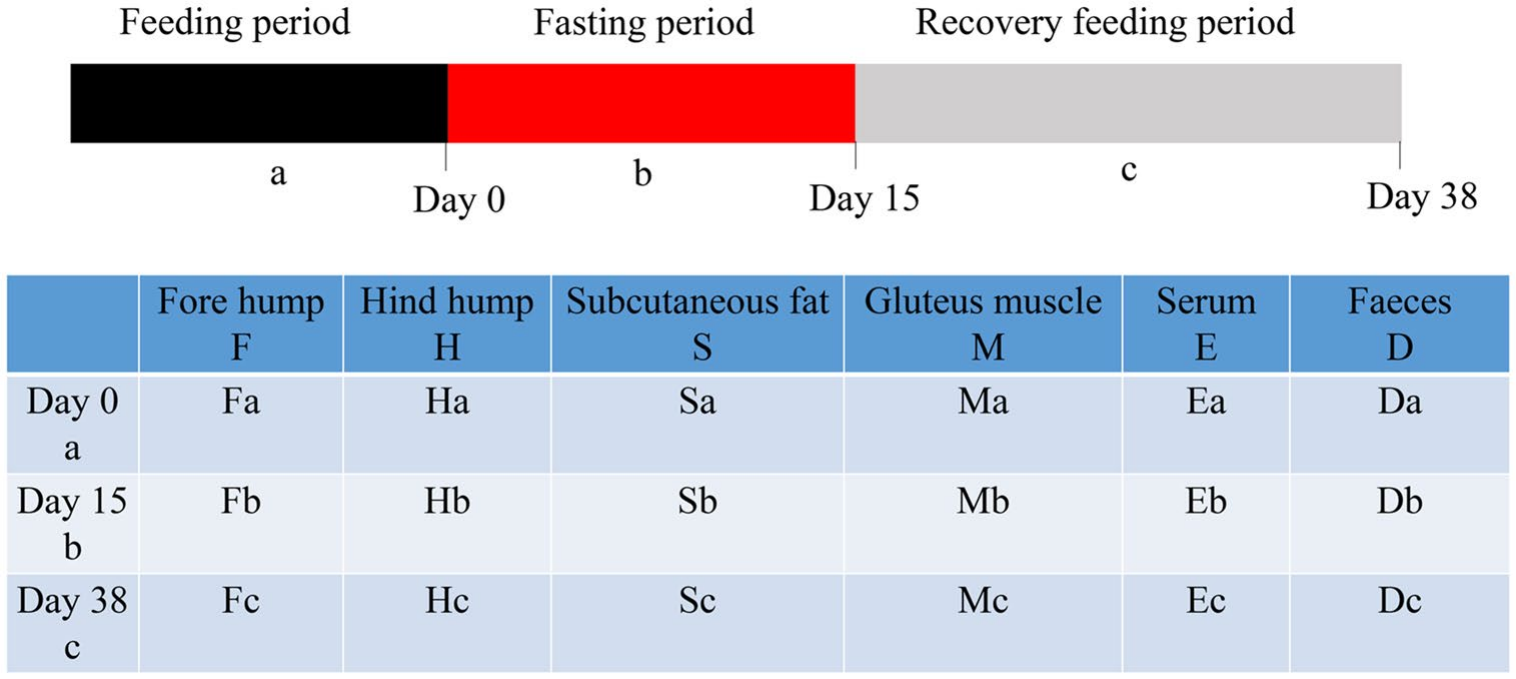
Experimental design and sample collection scheme.

We used 6 male domestic Bactrian camels (4 years old) that were born in the same captive environment and trained to meet the needs of the experiment. These camels were kept in individual pens. Camels ate and drank freely for a long time, mainly including alfalfa and corn (dry matter 28.75 g/100 g, protein 4.46 g/100 g, calcium 0.45 g/100 g, phosphorus 0.07 g/100 g, carotene 4.4 mg/100 g), which lasted until day 0. Before day 0 was the feeding period (a), 1 to 14 days was the fasting period (b), no water and food, and 14 to 38 days was the recovery feeding period (c), the diet was the same as that of stage a. Experiments and sample collection were carried out on the 0th day, 14th day and 38th day (Fig. 8). The following samples were collected: fore hump (F), hind hump (H), subcutaneous fat (S), biceps femoris muscle (M), serum (E) and faeces (D). Tissue samples were biopsied using a 6 mm circular punch. Each area was shaved and aseptically prepared for biopsy. Subcutaneous fat was obtained from the hindlimb region over the gluteus muscles. Muscle samples were obtained with the 6 mm punch via a short stab incision through the dermis over the lateral aspect of either the left or right biceps femoris muscle. Tissue samples were placed in a 2 ml freezing tube, immediately immersed in liquid nitrogen, and then stored at -80 °C until the subsequent test. After camels defecated, faecal samples were quickly collected and immediately preserved in liquid nitrogen and then stored at − 80 °C until DNA extraction. Whole blood was collected from the jugular vein into vacutainers without anticoagulant. For clinical chemistry panels (CCPs), blood without anticoagulant was centrifuged at 3000× g at 4 °C for 15 min to obtain serum. The separated serum was immediately analysed by an automatic biochemical analyser (Hitachi (China) Company, Beijing, China). Including insulin, glucose levels, leptin levels, adiponectin levels, nonesterified fatty acid and triglyceride.

### Reverse transcription and quantitative real-time polymerase chain reaction (qRT-PCR)

To clarify the metabolic state of camels during starvation, we selected 11 genes that are involved in the insulin metabolism pathway. qRT-PCR was conducted using a LineGene 9600 Plus fluorescence quantitative polymerase chain reaction (PCR) detection system (Hangzhou Langji Scientific Instrument Co., Ltd., Hangzhou, China) and ChamQ SYBR COLOR qPCR Master Mix (2X) (Vazyme Biotech Co., Ltd., Nanjing, China). RNA (F, H, S, M) melted at 4 °C immediately after it was removed from the freezer at − 80 °C. Then, the reaction solution was prepared in 0.2 ml PCR tubes, which were incubated at 37 °C for 15 min, incubated at 98 °C for 5 min for denaturation, and incubated at 4 °C. The following annealing protocol was applied: 1 cycle at 95 °C for 3 min, followed by 35 cycles at 95 °C for 30 s, 52 °C for 30 s, and 72 °C for 40 s (Supplementary Table 1), and the dissolution curve was obtained. Threshold cycle (CT) values were used to perform the 2 − ΔΔCt method and calculate the relative expression levels of genes (internal reference gene: β-actin)^54^.

### Insulin tolerance tests and challenges

Intravenous injection of 0.4 U/kg^55^ insulin (Novolin, Novo Nordisk) was conducted on 6 camels. Then, a blood glucose metre (Accu-Chek Active, Roche Diagnostics, Basel, Switzerland) was used to measure blood glucose levels. The separated serum was packed in a 200 µl sterile tube and stored at − 80 °C for later verification of blood glucose metre readings. When the blood glucose concentration was lower than 2.5 mmol/l, a 50% glucose solution was immediately injected intravenously, and the tolerance test was completed. This experiment was carried out on days 0, 15 and 38, respectively.

### Metabolomics analysis

Broad metabolite profiling of serum (E) was performed using liquid chromatography mass spectrometry (LC–MS; Agilent 1290 coupled to an Agilent 6545 MS System, Agilent, Waldbronn, Germany) and LC-tandem MS (LC–MS/MS; Agilent 1100 HPLC, API 4000, Applied Biosystems, Darmstadt, Germany). The Agilent 6545A QTOF mass spectrometer was under the control of the control software (LC/MS Data Acquisition, version B.08.00) based on the Auto MS/MS mode to perform primary and secondary mass spectrometry data acquisition with a mass scanning range of m/z 50–1100. The positive and negative ion modes are used for collection. The parameters of the electrospray ionization (ESI) ion source were set as follows: dry gas temperature (GasTemp): 320 °C; nitrogen flow (GasFlow): 8 Lpm; sheath gas velocity (SheathGasFlow): 12 L/min; sheath temperature (SheathGasTemp): 350 °C; and capillary voltage (VCap): 3500 V (negative ion mode), 4000 V (positive ion mode).

### 16S rRNA sequencing

Microbial genomic DNA was extracted from faecal samples (D) using a Mag-Bind Soil Kit (Omega, M5635). After the genomic DNA extraction was completed, 1% agarose gel electrophoresis was used to detect the quality of the extracted DNA, and a NanoDrop2000 was used to determine the concentration and purity of the DNA. The V3-V4 hypervariable region of the 16S rRNA gene was amplified by PCR with 338F (5′-ACTCCTACGGGAGGCAGCAG-3′) and 806R (5′-GGACTACHVGGGTWTCTAAT-3′). After mixing the PCR products of the same sample, a 2% agarose gel was used to recover PCR products, an AxyPrep DNAGel Extraction Kit (Axygen Biosciences, Union City, CA, USA) was used to purify the recovered products, 2% agarose gel electrophoretic detection was used, and a QuantusTM Fluorometer (Promega, USA) was used to detect and determine the amount of the recovered products. A NEXTFLEX Rapid DNA-Seq Kit was used to build the library. The MiSeq PE300 platform (Illumina) was used for sequencing.

### Statistical analysis

GraphPad Prism version 8.0.2 was used to analyse the serum biochemical indexes and qRT-PCR data. MetaboAnalyst4.0 (http://www.metaboanalyst.ca) software was used to analyse the differences in and enrichment of metabolites. Microbial diversity was analysed using the R 3.6.3 software package (https://www.r-project.org). The sequencing data from this study were deposited in the NCBI Sequence Read Archive (SRA) under accession number PRJNA714601.

### Ethics statement

All experimental design and procedures of this study was carried out in compliance with the ARRIVE guidelines (https://arriveguidelines.org). The procedures and protocols were approved by the animal care committee of the Camel Protection Association of Inner Mongolia.

## Supplementary Information

Supplementary Information.
