# Supplementary material for: Reversible insulin resistance helps Bactrian camels survive fasting
Source: Sci Rep. 2021 Sep 22;11:18815. doi: 10.1038/s41598-021-98234-y (PMC8458433; doi:10.1038/s41598-021-98234-y)
Supplement: Supplementary file 1 — Supplementary Information. [file 41598_2021_98234_MOESM1_ESM.pdf]

Reversible insulin resistance helps Bactrian camels survive fasting

Fucheng Guo[1], Rendalai Si[1,2], Quanyun Li[1], Le Hai[1], Li Yi[1], Jing He[1], Liang Ming[1] and Rimutu Ji[1,2]

[1]Key Laboratory of Dairy Biotechnology and Bioengineering, Ministry of Education, College of Food Science and Engineering, Inner Mongolia Agricultural University, Hohhot, 010018, China; guofucheng1101@163.com (F.G.); liquanyunimau@163.com (Q.L.); sirendalai_imau@163.com (R.S.); haileaijia@163.com (L.H.); yili_imau@163.com (L.Y.); hejing1409@163.com (J.H.).

[2]Camel Research Institute of Inner Mongolia, Alxa, 737300, China

Correspondence: bmlimau@163.com (Liang Ming); yeluotuo1999@vip.163.com (Rimutu Ji).

Supplementary Table 1. Real time PCR reaction conditions.

Gene	Primer Name	Sequence（5'--3'）	Thermal profile
PIK3CB	3-PIK3CB-F	CTGCAGAGAGAATTTCCCAC	95℃ for 3 min ×1cycle；
	3-PIK3CB-R	GCATATTCTCGCACGTACTG	95℃ for 30s，52℃ for 30s，72℃ for 40s，×35cycles；
PIK3R1	4-PIK3R1-F	GAAGATGACGAGGACTTGCC	95℃ for 3 min ×1cycle；
	4-PIK3R1-R	GTCTTGTTGATGACGCAGTG	95℃ for 30s，52℃ for 30s，72℃ for 40s，×35cycles；
SLC2A4	5-SLC2A4-F	TGAACTATGTCTCCATCGTG	95℃ for 3 min ×1cycle；
	5-SLC2A4-R	CCATAGCATCCGCAACATAC	95℃ for 30s，52℃ for 30s，72℃ for 40s，×35cycles；
IRS1	10-IRS1-2F	ATCAGAGCTACGTGGACACC	95℃ for 3 min ×1cycle；
	10-IRS1-2R	CAGAGGTCGTGGAGGATGAG	95℃ for 30s，52℃ for 30s，72℃ for 40s，×35cycles；
MTOR	11-MTOR-F	TGGAATATGCCATGAAACAC	95℃ for 3 min ×1cycle；
	11-MTOR-R	CCTTGTTGGTGTCCATCTTC	95℃ for 30s，52℃ for 30s，72℃ for 40s，×35cycles；
AKT3	12-AKT3-F	CAACCAGGATCATGAGAAAC	95℃ for 3 min ×1cycle；
	12-AKT3-R	TACATCTTGCCAGTTTACTC	95℃ for 30s，52℃ for 30s，72℃ for 40s，×35cycles；
AKT1	13-AKT1-2F	AATGGTCGGCAGTTTTTCTT	95℃ for 3 min ×1cycle；
	13-AKT1-2R	TGGCTGCAGAAGTCCTCAAC	95℃ for 30s，52℃ for 30s，72℃ for 40s，×35cycles；
GSK3A	14-GSK3A-F	CATCTTCCCTGGGGATAGTG	95℃ for 3 min ×1cycle；
	14-GSK3A-R	GGGATTTGAAAACCTTTGTC	95℃ for 30s，52℃ for 30s，72℃ for 40s，×35cycles；
PTEN	15-PTEN-F	TCAATTGTAATGACTGCTCC	95℃ for 3 min ×1cycle；
	15-PTEN-R	ACATGAAGCATCCACAGCAG	95℃ for 30s，52℃ for 30s，72℃ for 40s，×35cycles；
TSC2	16-TSC2-3F	CCTCGGACATCAACAACA	95℃ for 3 min ×1cycle；
	16-TSC2-3R	TTAGCCTCACCTGGACTT	95℃ for 30s，52℃ for 30s，72℃ for 40s，×35cycles；
INSR	18-INSR-F	ACAGACCCTTCGAGAAAGTG	95℃ for 3 min ×1cycle；
	18-INSR-R	GGCATCTTGGTTGCAAGC	95℃ for 30s，52℃ for 30s，72℃ for 40s，×35cycles；
β-actin	β-actin-F	GGACTTCGAGCAGGAGATGG	95℃ for 3 min ×1cycle；
	β-actin-R	AGGAAGGAGGGCTGGAAGAG	95℃ for 30s，52℃ for 30s，72℃ for 40s，×35cycles；

Supplementary Table 2. Representative Kyoto Encyclopedia of Genes and Genomes (KEGG) pathways of the 365 differentially expressed metabolites (DEMs).

pathway	Description	Count	P adjust	Enrichment Fold
ko00121	Secondary bile acid biosynthesis	11	3.88E-09	16.40337
ko05230	Central carbon metabolism in cancer	10	6.39E-08	14.50912
ko04974	Protein digestion and absorption	10	5.41E-07	11.42208
ko00360	Phenylalanine metabolism	11	2.78E-06	8.201686
ko00970	Aminoacyl-tRNA biosynthesis	8	0.000142	8.25904
ko00220	Arginine biosynthesis	5	0.001241	11.67038
ko00471	D-Glutamine and D-glutamate metabolism	4	0.001241	17.89459
ko04976	Bile secretion	12	0.001848	3.681172
ko00250	Alanine, aspartate and glutamate metabolism	5	0.002141	9.586386
ko00630	Glyoxylate and dicarboxylate metabolism	7	0.002141	6.06107
ko02010	ABC transporters	10	0.002141	4.194044
ko00627	Aminobenzoate degradation	8	0.002157	5.052589
ko04978	Mineral absorption	5	0.002157	9.255821
ko00240	Pyrimidine metabolism	7	0.002391	5.693732
ko00260	Glycine, serine and threonine metabolism	6	0.00327	6.442051
ko00660	C5-Branched dibasic acid metabolism	5	0.003812	7.894671
ko01040	Biosynthesis of unsaturated fatty acids	6	0.004415	5.964862
ko04727	GABAergic synapse	3	0.004443	17.89459
ko04721	Synaptic vesicle cycle	3	0.010582	13.42094
ko00460	Cyanoamino acid metabolism	5	0.010835	5.964862
ko00940	Phenylpropanoid biosynthesis	6	0.010835	4.880342
ko00120	Primary bile acid biosynthesis	5	0.012058	5.711038
ko00623	Toluene degradation	5	0.012058	5.711038
ko00330	Arginine and proline metabolism	6	0.020789	4.12952
ko00350	Tyrosine metabolism	6	0.020789	4.12952
ko00680	Methane metabolism	6	0.028908	3.834554
ko00473	D-Alanine metabolism	2	0.029849	17.89459
ko05033	Nicotine addiction	2	0.039807	15.33822
ko00230	Purine metabolism	6	0.04469	3.426623
ko00960	Tropane, piperidine and pyridine alkaloid biosynthesis	5	0.045879	3.947335
ko04724	Glutamatergic synapse	2	0.045879	13.42094
ko05030	Cocaine addiction	2	0.045879	13.42094

Supplementary Table 3. Faecal (D) sample sequencing information statistics. a: feeding period, b: fasting period, c: recovery feeding period.

Sample	Seq_num	Base_num	Mean_length	Min_length	Max_length
D_b_3	42768	18694278	437.109	325	463
D_a_1	89070	38722075	434.7376	269	501
D_c_4	62079	26935274	433.887	266	457
D_c_5	60776	26388098	434.1862	286	464
D_b_1	61232	26780325	437.3583	290	469
D_c_2	61180	26503438	433.2043	276	458
D_b_4	54457	23843349	437.8381	285	452
D_a_3	85310	36938523	432.9917	276	481
D_b_5	56683	24464813	431.6076	272	511
D_a_5	73829	32065311	434.3186	265	513
D_b_6	55277	23935653	433.0129	316	473
D_a_2	72267	31147989	431.0126	294	476
D_b_2	50759	22061569	434.6336	307	468
D_c_3	55129	23931091	434.0926	219	490
D_c_1	58914	25495095	432.751	338	452
D_a_6	72607	31409647	432.5981	270	470
D_a_4	70493	30545084	433.3066	274	504
D_c_6	66853	28938792	432.872	280	452

Supplementary Table 4. Comparison of functional pathway between Db and Da & Dc (D: Faecal, a: feeding period, b: fasting period, c: recovery feeding period, $P < 0.01$).

Pathway level1	Pathway level2	Pathway level3	Level3 Description
Environmental Information Processing	Signaling Molecules and Interaction	ko04030	G protein-coupled receptors
Human Diseases	Metabolic Diseases	ko04940	Type I diabetes mellitus
Environmental Information Processing	Signal Transduction	ko04011	MAPK signaling pathway - yeast
Metabolism	Metabolism of Terpenoids and Polyketides	ko00906	Carotenoid biosynthesis
Metabolism	Biosynthesis of Other Secondary Metabolites	ko00945	Stilbenoid, diarylheptanoid and gingerol biosynthesis
Metabolism	Xenobiotics Biodegradation and Metabolism	ko00983	Drug metabolism - other enzymes
Human Diseases	Neurodegenerative Diseases	ko05010	Alzheimer's disease
Cellular Processes	Cell Growth and Death	ko04210	Apoptosis
Metabolism	Amino Acid Metabolism	ko00310	Lysine degradation
Metabolism	Metabolism of Cofactors and Vitamins	ko00670	One carbon pool by folate
Metabolism	Biosynthesis of Other Secondary Metabolites	ko00524	Butirosin and neomycin biosynthesis
Metabolism	Lipid Metabolism	ko00592	alpha-Linolenic acid metabolism
Metabolism	Metabolism of Terpenoids and Polyketides	ko00281	Geraniol degradation
Unclassified	Metabolism	-	Energy metabolism
Metabolism	Metabolism of Terpenoids and Polyketides	ko01053	Biosynthesis of siderophore group nonribosomal peptides
Metabolism	Lipid Metabolism	ko00565	Ether lipid metabolism
Human Diseases	Infectious Diseases	ko05142	Chagas disease (American trypanosomiasis)
Metabolism	Metabolism of Cofactors and Vitamins	ko00830	Retinol metabolism
Unclassified	Cellular Processes and Signaling	-	Cell division
Human Diseases	Infectious Diseases	ko05120	Epithelial cell signaling in Helicobacter pylori infection
Genetic Information Processing	Folding, Sorting and Degradation	BR:ko04121	Ubiquitin system
Metabolism	Biosynthesis of Other Secondary Metabolites	ko00521	Streptomycin biosynthesis
Organismal Systems	Endocrine System	ko04614	Renin-angiotensin system

Metabolism	Metabolism of Terpenoids and Polyketides	ko00523	Polyketide sugar unit biosynthesis
Metabolism	Lipid Metabolism	ko01040	Biosynthesis of unsaturated fatty acids
Organismal Systems	Environmental Adaptation	ko04712	Circadian rhythm - plant
Metabolism	Metabolism of Other Amino Acids	ko00430	Taurine and hypotaurine metabolism
Unclassified	Metabolism	-	Nucleotide metabolism
Metabolism	Metabolism of Other Amino Acids	ko00471	D-Glutamine and D-glutamate metabolism
Human Diseases	Infectious Diseases	ko05146	Amoebiasis
Unclassified	Cellular Processes and Signaling	-	Other ion-coupled transporters
Metabolism	Xenobiotics Biodegradation and Metabolism	ko00930	Caprolactam degradation
